# Photolithography Fabricated Spacer Arrays Offering Mechanical Strengthening and Oil Motion Control in Electrowetting Displays

**DOI:** 10.3390/s20020494

**Published:** 2020-01-15

**Authors:** Yingying Dou, Lin Chen, Hui Li, Biao Tang, Alex Henzen, Guofu Zhou

**Affiliations:** 1 Guangdong Provincial Key Laboratory of Optical Information Materials and Technology & Institute of Electronic Paper Displays, South China Academy of Advanced Optoelectronics, South China Normal University, Guangzhou 510006, China; douyingying@scnu.edu.cn (Y.D.); 2016022681@m.scnu.edu.cn (L.C.); alex-henzen@scnu.edu.cn (A.H.); 2 National Center for International Research on Green Optoelectronics, South China Normal University, Guangzhou 510006, China; 3 College of Mechatronics and Control Engineering, Shenzhen University, Nanhai Ave 3688, Shenzhen 518060, China; huili@szu.edu.cn; 4 Shenzhen Guohua Optoelectronics Tech. Co. Ltd., Shenzhen 518110, China; 5 Academy of Shenzhen Guohua Optoelectronics, Shenzhen 518110, China

**Keywords:** electrowetting display, oil motion, spacer arrays, mechanical strength, optoelectronic performance

## Abstract

Introducing spacers into pixelated electrowetting displays (EWDs) normally gives mechanical strengthening, while bringing undesired disturbance of water/oil interfacial dynamics. Hence, spacer array is a key pixel structure needs careful consideration in the design and fabrication of electrowetting displays. Here, we propose a spacer array, which is designed standing on the junction of adjacent pixel walls, fabricated by photolithography. The spacer array provides mechanical strength enhancement and reliable oil motion controllability. By optimizing the spacer distribution density, the EWD device may achieve 28% increase in open ratio (white area fraction) and withstand 60 N/mm^2^ pressure. This design of spacer array reasonably solves the contradiction between mechanical strength enhancement and optoelectronic performance in EWDs, providing potential applications in oil–water two-phase microfluidic devices.

## 1. Introduction

Electronic paper-like (e-paper) displays have attracted more and more attentions because of their natural light reflection property, which gives unique advantages in portable reading, electronic shelf-labels, tablets, billboards, etc. [1,2,3] Electrowetting display (EWD) reported in 2003 is a very promising display technology, which enables full color and video contents playing on e-paper devices for the first time [4]. Typically, the optical active area of EWD is covered by hydrophobic Teflon coating [5] surrounded by relatively hydrophilic walls fabricated by photolithography. Non-polar colored oil covers hydrophobic area while the conductive aqueous solution on top is insulated inside the pixel. Once a bias voltage is applied, the electrostatic force attracts water to the insulating layer, which induces the rupture of oil film and the following motion dominated by dewetting process [6,7,8]. The oil motion models are determined by the oil/water interfacial properties and pixel geometries [4,9,10,11,12]. However, the optical response of EWD after oil film rupture is generally expected following Young–Lippmann equation [13]:

(1) $$\cos\theta ={\cos\theta }_{0}+{\mathrm{cU}}^{2}/2\sigma ,$$

where, the subscripts θ and θ_0_ represent the contact angles of a conductive droplet on a hydrophobic solid surface driven at applied voltage of U or 0 V, respectively. σ is the interfacial tension between oil phase and conductive liquid phase. c is the capacitance per unit area between the conductive liquid phase and the bottom electrode; and U is the applied voltage.

After the switching-on process with a bias voltage, the colored oil in EWD display is repelled to be an oil droplet at the side or the corner of a pixel with the decreasing θ (Equation (1)). As a result, oil could be driven to move to various directions because of the high symmetry of square pixels, leading to random oil motion states and thus non-uniform optical view [14]. Here, colored oil motion is used to describe the movement of oil/water interface. Consequently, various methods have been reported to improve the oil motion controllability. For example, pixel shapes with reduced symmetry have been designed to guide oil motion via capillary force difference, including rectangle, triangle, and trapezoid pixels [14,15]. Meanwhile, an extra obstacle in a pixel also could decrease the symmetry of square pixels. For example, an extra pin structure (EPS) or hydrophilic/hydrophobic microparticles in pixels have induced the reorganization of oil film to different thickness distributions during the filling process, thus resulting in improved oil motion controllability after the oil film rupturing from the thinnest point [10,16]. Besides, hydrophilic–hydrophobic or geometry staircase has also been introduced to pixels to guide oil/water interface motion. Giraldo et al. reported pixels with hydrophilic patch or geometry staircase to obtain the thinnest oil film thickness near the hydrophilic patch or above the geometry staircase, leading to the oil motion to the opposite corner of the patch or the staircase at switching-on process [17]. On the other hand, a hydrophobic cladding area placed on insulator surface and patterned at one corner of the square pixel was designed to attract oil, resulting in oil droplet moving to this cladding area after switching-on [18]. Moreover, asymmetric electric field in space or in time could also induce a guided oil motion in pixel. Certain notch area on the electrode has been designed to control oil motion via asymmetric electric field distribution [14]. In general, this oil motion controllability improves with increasing notch size. Electric driving waveforms have also been employed to control oil motion via electric field induced force differentiation [6]. Precise design and understanding of different parameters is one of the key points for optofluidic device design and practical application.

On the other hand, oil droplet formed with applied voltage reduces the distance between the top electrode and oil, which increases the possibility of oil droplet attaching and then staying on the top electrode, leading to the display becoming invalid, especially during a pressure test to the display or bending a flexible display. Hence, spacer structure between the cover and bottom substrates is significant for the gap stability, beneficial for the further stability of three phase contact line. Spacer arrays improve the mechanical strength of EWD. However, spacers formed in EWD easily disturb the oil motion of pixels [19]. Hence, a spacer structure owning enough mechanical strength and good oil motion controllability is needed for EWD display, which is a big challenge.

Meanwhile, photolithography is proved to be a good method to fabricate accurate microstructures, especially for microfluidic devices [20] and micro-electromechanical systems (MEMS) [21]. Photolithography not only provides the advantages of good accuracy controllability [22] and design adjustment, but also provides a way to fabricate 3D structures with varied properties by several steps of photolithography [23].

In this work, novel spacer arrays (SAs) standing on the junction of EWD pixels have been designed and precisely fabricated by photolithography. The mechanical strength and oil motion controllability enabled by the spacer array introduced into the EWD device have been studied. The influences of the distribution density and length scale of the spacer array are investigated. 

## 2. Experimental Section 

### 2.1. Chemicals and Materials

Indium tin oxide (ITO, 25 nm) coated glass (0.7 mm thick) with sheet resistance of 100 Ω/sq (Guangdong Jimmy Glass Technology Ltd., Foshan, China) was used as the electrode substrate and the cover plate. Amorphous fluoropolymer AF 1600X (Chemours Chemical Co., Ltd., Shanghai, China) was used to prepare the insulator layer. Pixel walls and the spacer arrays were prepared by two step photolithography processes using two different negative photoresists (HN resist for pixel wall and SUN-1901N resist for spacer, Suntific, Weifang, China). Deionized (DI) water was prepared by a water purification system (Yili, Guangzhou, China) and used as the conductive liquid without further treatment. A purple dye was dissolved in decane (C_10_H_22_) at concentration of 0.21 mol/L and used as the non-polar oil liquid. EWD was sealed with a pressure sensitive adhesive (PSA). 

### 2.2. EWD Fabrication

**Figure 1:**
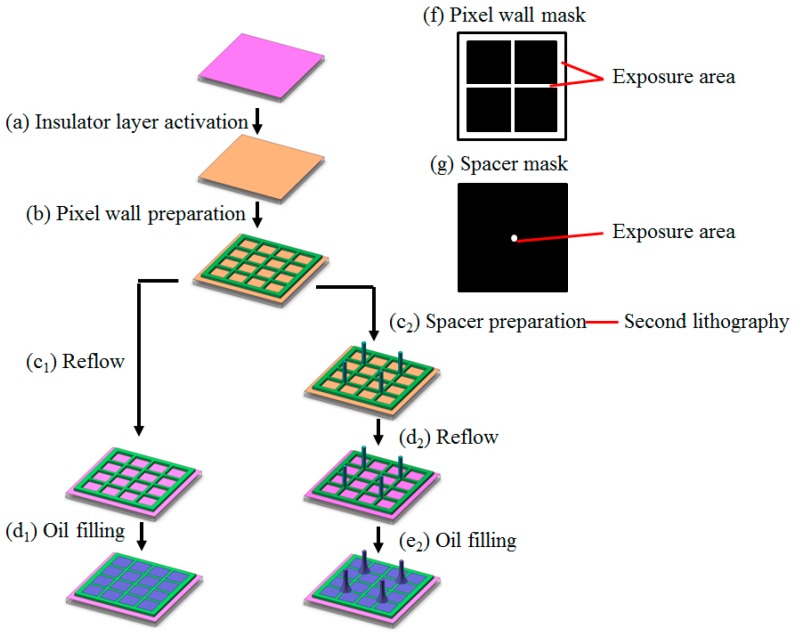
Schematic of the fabrication process of the backplane of electrowetting display (EWD) devices, from the process of insulator layer activation (**a**) to the process of oil filling (**d_1_**,**e_2_**). After the insulator layer activation (**a**), pixel wall layer is prepared (**b**) using the pixel wall photolithography mask (**f**), along with reflow process (**c_1_**) leading to hydrophobic recovery of insulator surface and then the oil filling (**d_1_**) for pixels without spacer arrays (No-SAs pixels). Meanwhile after the pixel wall fabrication is the spacer layer preparation (**c_2_**) using spacer mask (**g**) for pixels with spacer arrays (SAs pixels). After the reflow process (**d_2_**), the SAs pixels could be oil filled (**e_2_**).

After the ITO glass cleaning process in a cleaning line (KJD-7072ST, KEJINGDA Ultrasonic Equipment Co., Ltd., Shenzhen, China), amorphous fluoropolymer solution was spin-coated on the ITO surface at 1400 rpm for 60 s using a spin coater (KW-5, Institute of Microelectronics Chinese Academy of Sciences, Beijing, China). The fluoropolymer film with a thickness of around 850 nm was obtained after drying on a hotplate at 85 °C for 5 min and then in an oven at 185 °C for 2 h, as the insulator layer. The hydrophobic AF 1600X surface was then treated to be hydrophilic using a reactive ion etching (RIE) machine (ME-6A, Institute of Microelectronics Chinese Academy of Sciences, Beijing, China) with oxygen plasma at 5 W for 10 s, which is the insulator layer activation (Figure 1a). HN photoresist film was spin-coated onto the hydrophilic AF 1600X surface using a smart spin-coater (SC100, Best Tools, Jiangsu LEBO Science Instruments, Co., Ltd., Jiangyin, China), and then patterned with a standard photolithography process using an aligner instrument (URE-2000/35, Institute of Optics and Electronics, Chinese Academy of Sciences, Chengdu, China) to form the pixel wall (Figure 1b) with the height of 5.6 μm and width of 15 μm, while the periodic length of the square pixel was 165 μm. The photolithography mask of pixel wall was as shown in Figure 1f. Thick photoresist (SUN-1901N) coating and photolithography process to form the spacer arrays (Figure 1c_2_) were carried out afterwards. Different spacer height (60 μm, 40 μm, and 20 μm) was obtained by adjusting the coating and photolithography parameters. Spacer arrays with different densities were fabricated by a special designed photolithography mask (Figure 1g) with the density (No._spacer_:No._pixel_) of 1:1 (1 spacer per 1 pixel), 1: 4 (1 spacer per 4 pixels), 1:16 (1 spacer per 16 pixels), and 1:64 (1 spacer per 64 pixels). A thermal reflow process at 200 ± 5 °C for 2 h in an oven (5FG-01B, Huangshi, China) was applied to restore the hydrophobicity of the fluoropolymer layer after the pixel wall (Figure 1c_1_) or spacer array (Figure 1d_2_) preparation. The oil filling (Figure 1d_1_,e_2_) and device assembly process was applied to form the integrated EWDs [16,24].

### 2.3. Wettability Characterization

A contact angle measurement system (POWEREACH, Shanghai Zhongchen Digital Technology Apparatus Co., Ltd., Shanghai, China) with a flat needle was used to measure the contact angles of the pixel wall material and spacer material, which included the water contact angle in air atmosphere (air/water contact angle) and the oil (0.21 mol/L purple dye in decane) contact angle in water atmosphere (oil/water contact angle). Advancing and receding contact angles were measured by adding or removing liquid from the water drop deposited on the pixel wall film surface or the spacer film surface. The volume of the water droplet for each measurement was 5 μL.

### 2.4. Mechanical Strength Measurement

A digital pull-push meter (HP-200, Handpi instruments, Leqing, China) was used to measure the pressure on displays. The diameter of the testing probe was 2 mm, indicating a contact area of 3.14 mm^2^ between the probe and the display. The pressure was kept increasing by a hand wheel and stopped when the oil film defect on display appeared.

Videos of the pressure applying process were recorded by a camera (AT-X M100 AF PRO, Tokina, Japan) with the magnification of 1.68. Then photos before pressing, pressing at a certain force (60 N for display without SAs, and 110 N for displays with different spacer densities) and after pressing were cut from videos. The pressure was applied by manual pressing during video recording.

### 2.5. Switching Behavior of Electrowetting Display

A high-speed camera (Phantom MIRO M110, Wayne, USAs) was used to observe the oil motion during switching process of pixels at a recording frame rate of 1600 fps or 2800 fps. The voltage was applied via a high voltage source (GWINSTEK PSW 800-1.44, Taiwan, China). Oil motion in pixels with or without SAs was compared, while the influences of spacer densities (1:1, 1:4, and 1:16), and spacer heights (60 μm, 40 μm, and 20 μm) were also tested. The frame before the oil rupture was taken as the starting point (0 ms).

## 3. Results and Discussion

### 3.1. Fabrication of Spacer Arrays

In this work, spacer arrays (SAs) were fabricated on the junction of near four pixels of EWD device, which needs a second photolithography process (Figure 1c_2_). Pixel wall preparation is in the first photolithography process (Figure 1b) after the hydrophilic activation to the insulator layer (Figure 1a), using the pixel wall mask (Figure 1f), while the mask of spacer is as shown in Figure 1g. When the insulator was thermo reflowed back to hydrophobic (Figure 1c_1_,d_2_), oil could be filled into pixels (Figure 1d_1_,e_2_), to finish the fabrication of EWD display with assembly.

### 3.2. Mechanical Strength Enhancement by Spacer Arrays

**Figure 2:**
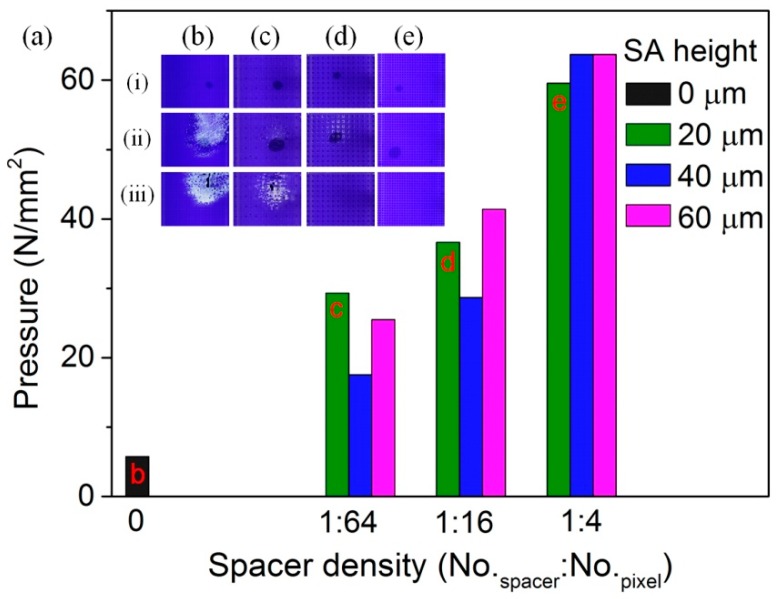
(**a**) The pressure at the oil film rupture point of EWD displays with different spacer densities (0, 1:64, 1:16, and 1:4). The spacer height is 20 μm, 40 μm, and 60 μm. The letters b, c, d, and e indicate the spacer density in graphs (**b**–**e**), respectively. (**b**–**e**) The pictures taken by camera showing the display pixels before pressing (i), during pressing (pressure, ii), and after pressing (iii). The pressure for the pictures taken at the pressing state (ii) is 60 N (**b**) and 110 N (**c**–**e**). The spacer density is 0 (**b**), 1:64 (**c**), 1:16 (**d**), and 1:4 (**e**). The spacer height of (**c**–**e**) is 20 μm. The black dots in the inserted graphs are the tip shadows of the meter on display.

The fabricated spacer arrays (SAs) of electrowetting displays help enhance the mechanical strength. As shown in Figure 2a, the application of spacer arrays could increase the strength of displays greatly with the spacer height of 20 μm, 40 μm, and 60 μm. Here, the pressure for oil film rupture (force per unit area, N/mm^2^) was used to characterize displays’ strength. The rupture pressure indicates the pressure with which the pixel white defect occurs due to the oil film rupture. The No-SAs electrowetting display could bear the pressure of 6 N/mm^2^, while displays with SAs became strong (≥18 N/mm^2^). The rupture pressure of EWDs with three SA heights increased with spacer density linearly, which means that EWD devices with larger spacer density obtained larger mechanical strength. Meanwhile, the height influence to mechanical strength was complex. The mechanical strength did not simply increase or decrease with spacer height: the middle height sample (40 μm) had the lowest strength with low density (1:16 and 1:64), while lowest spacer sample (20 μm) had the smallest strength with high SA density (1:4).

To explore the density influence more directly, graphs of EWDs with different spacer densities (0, 1:64, 1:16, and 1:4) before pressing (i), during pressing (ii), and after pressing, (iii) were inserted in Figure 2, with the spacer height of 20 μm. As the pressure was applied by hand, the contact area was not accurate, as the black dots in these graphs (Figure 2b–e, i–ii) showing. Hence, pressure (N) was used to characterize the different mechanical strength of SAs displays. Pressure of 60 N was applied on No-SAs EWD display (Figure 2b), and the white pixel defects appeared during and after pressing with a large defect area. Meanwhile, a pressure of 110 N was applied on SAs EWDs with different spacer densities (1:64 in Figure 2c, 1:16 in Figure 2d, and 1:4 in Figure 2e). White pixel defects appeared for display with spacer density of 1:64 (Figure 2c), whose defect area was smaller than that in No-SAs display (Figure 2b); self-switching-on pixels were observed for display with spacer density of 1:16 (Figure 2d) during pressing, while self-switching-off occurred after pressing without white pixel defect; and no response and no white pixel defect was observed for display with spacer density of 1:4 (Figure 2e) during or after pressing. As a consequence, displays with spacer arrays performed larger mechanical strength than displayers without SAs, and larger SA density was more beneficial for the mechanical stability of EWDs.

### 3.3. Oil Motion Control of Spacer Arrays

#### 3.3.1. Spacer Density and Height

**Figure 3:**
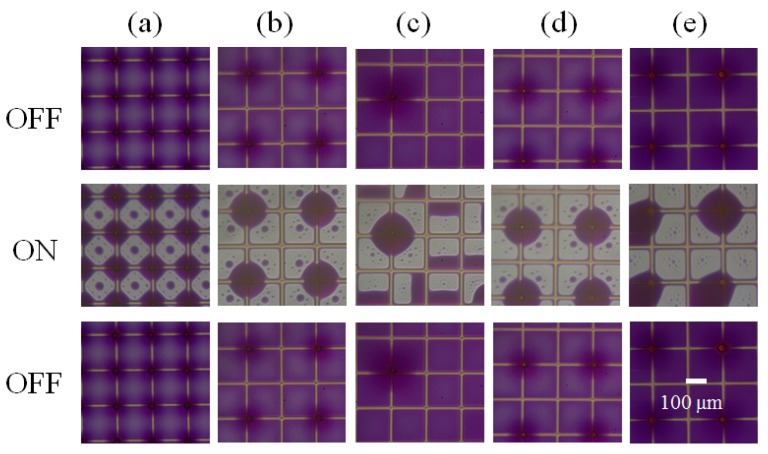
Graph shows the OFF, ON, and OFF states from up to bottom with applied voltage of 30 V. (1) Effect of spacer densities (number of spacers: number of pixels, (**a**) 1:1, (**b**) 1:4, and (**c**) 1:16 on oil motion and gathering behavior with the spacer height of 60 μm. (2) Effect of spacer heights ((**b**) 60, (**d**) 40, and (**e**) 20 μm) on oil motion and gathering behavior with the spacer density of 1:4.

The spacer arrays (SAs) designed and fabricated to stand on the junction of pixels show good controllability of oil motion, which is beneficial for the optical view of EWD. To search the suitable parameters of this controlling, the density of spacers varies with the density defined as the ratio of spacer number to pixel number (No._spacer_:No._pixel_). As shown in Figure 3a–c and Figure S1a–c, the spacer density was 1:1, 1:4, and 1:16, respectively, with the spacer height of 60 μm and the applied voltage was 30 V at the “ON” state. For the 1:1 ratio (adjacent distribution), each junction was occupied by a spacer, as shown in Figure 3a, resulting in the concave oil film in each pixel with the thinnest point in the middle of a pixel. Thus, when a bias voltage was applied, the oil film rupture occurred at the center of each pixel forming four small oil droplets, which move and aggregate to the spacer arrays. The satellite droplet left in the pixel’s center is caused by the Rayleigh Plateau mechanism when a film (filament) ruptures suddenly induced by an extra force [25]. When the spacer density decreased to 1:4 (interphase distribution), as shown in Figure 3b, there was exactly one spacer standing on the junction of four pixel walls connecting four neighboring pixels. Under this situation, the oil film in each pixel was arranged as shown at the “OFF” state (Figure 3b), and ruptured from the opposite diagonal direction of the spacer at the switching-on process and then oils from four neighboring pixels gathered along one spacer forming a huge droplet. When the spacer density decreased further to less than 1:4, for instance 1:16 in Figure 3c, the spacer would gather oil from neighboring four pixels, without influencing the rest pixels. In this case, the spacer affected pixels acted like the interphase SAs pixels, while the pixels without spacer arrays (No-SAs pixels) were not influenced by spacer at all. Therefore, pixels with fully adjacent (1:1) or fully interphase (1:4) spacer distributions could achieve uniform oil motion control. While with lower spacer density, pixels could be partly controlled with different oil motion to obtain optical difference. The white area fraction (WA) at “ON” state was affected by SA density (Figure S1a–c): the highest SA density obtained the lowest WA (0.46 ± 0.00); the density of 1:16 owned the middle WA (0.58 ± 0.08); and the density of 1:4 owned the largest WA (0.64 ± 0.04). Combining the oil motion controllability and the white area fraction, the interphase spacer distribution with density of 1:4 was the best density choice.

Spacer height was tested with the best density of 1:4. The pixels with spacer height of 60 μm (Figure 3b and Figure S1b) and 40 μm (Figure 3d and Figure S1d) achieved a large oil droplet around spacer at 30 V, while the large oil droplet was in one pixel for the height of 20 μm (Figure 3e and Figure S1e). The reason was that high spacers with large pinning force were beneficial for oil droplet’s steady around spacer structure. Meanwhile, the white area fraction (WA) decreased with spacer height (Figure S1): 0.64 ± 0.04 for 60 μm height, 0.61 ± 0.03 for 40 μm height, and 0.56 ± 0.25 for 20 μm height. Considering the oil motion control and the white area fraction together, pixels with enough spacer heights (60 μm, 40 μm) have good oil motion control forming a large oil droplet around spacer array.

#### 3.3.2. Applied Voltage

**Figure 4:**
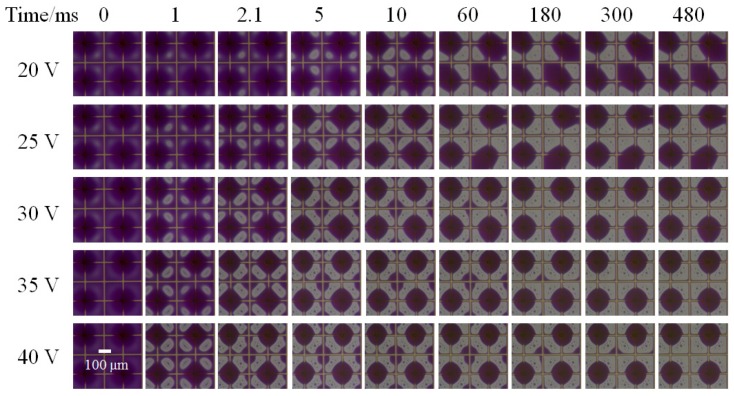
The oil–water state graphs with time at different voltages. The voltage is 20 V, 25 V, 30 V, 35 V, and 40 V from top to bottom. The spacer height is 60 μm and the spacer density is 1:4. The time of 0 ms is set as the time oil film rupture starting.

SAs pixels with the spacer density of 1:4 and thickness of 60 μm were taken as the example to show the oil motion with time at different voltages (Figure 4). From 20 to 40 V, all pixels had the oil rupture between spacers and the opposite pixel corner but away from the pixel center, and obtained a large oil droplet around the spacer along with a small oil droplet at the opposite corner at the beginning. At the oil rearrangement process after the coalescence of these two oil droplets, some of the formed large oil droplets move to one pixel at low voltage (20 V and 25 V), while all the droplets keep around the spacer structure at high voltage (30 V, 35 V, and 40 V). We believed the increasing electrostatic force with voltage was beneficial for oil being repelled to the spacer structure and then climbed high along spacers, which helped the droplet stick around spacers. Hence, high applied voltage is beneficial for improving oil motion controllability.

#### 3.3.3. Optical Performance of EWD with and without Spacer Arrays

**Figure 5:**
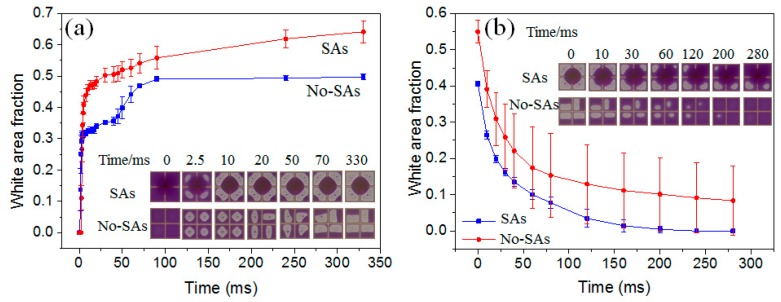
The white area fraction (WA) curve with time for pixels with or without spacer arrays (SAs) at 30 V. The switching process includes the opening process (**a**) and the closing process (**b**). The inserted graphs show the oil–water states at certain time. To show the clear influence of spacer on oil motion, four adjacent pixels are shown in the inserted graphs.

**Figure 6:**
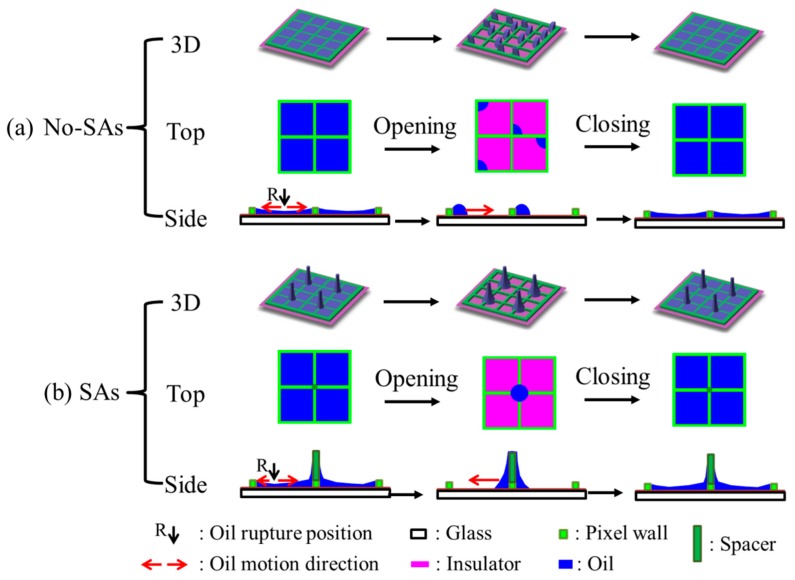
Schematic of the opening and closing processes of EWD devices without (**a**) and with (**b**) spacer arrays (SAs) at 3D, top, and side views. The pixels without SAs were named as No-SAs pixels, while pixels with SAs as SAs pixels. R position is position of oil film rupture occurs. Red arrows at the side view show the oil motion directions during opening or closing of EWD devices.

From the above parameter exploring, density of 1:4, enough height of 40 μm and 60 μm, enough applied voltage of 30 V, 35 V, and 40 V are the good parameters for oil motion control. Taking the optical performance (white area fraction and oil motion controllability) into account, we chose the pixels with spacer arrays (SAs pixels) with spacer density of 1:4 and height of 60 μm as an example to characterize the optical performance between SAs pixels and pixels without SAs (No-SAs pixels) in this section. During the dynamic switching-on process (Figure 5a), the white area fraction was observed to increase from fast to slow and then be stable with time. Oil rupture position of No-SAs pixels was at the center for these four pixels (Figure 5a and Figure 6a), while oil rupture of four SAs pixels occurred at the circle between SA and its opposite pixel corner at the same time (Figure 5a and Figure 6b). The oil rupture position was related to the under-fill condition of pixels. From the time scale in Figure 5a, oil rupture of No-SAs pixels occurred earlier than SAs pixels. The possible reason is that the oil film thickness of SAs pixels is higher than No-SAs pixels after filling because of the oil capture of spacer during filling (Figure 6). Following the oil film rupture process is the oil rearrangement process. In this process, oil moves to two sides first, forming two similar oil droplets in one No-SAs pixel. Then the two oil droplets coalesce to one large droplet. Due to the symmetry of square pixels, the oil motion uniformity was bad for No-SAs pixels. Meanwhile, one large oil droplet formed around the spacer structure for four SAs pixels together and one small droplet existed at the opposite corner to the spacer in each SAs pixel. At last, the small droplets in these four SAs pixels moved to the large oil droplet at the oil rearrangement process, leading to only one large oil droplet forming around the spacer structure for four SAs pixels. Due to the special spacer array design, the formed oil droplet around SA was regular, which improved the oil motion uniformity of SAs pixels. This process took a long time because of the long way to go, which resulted in the slower rearrangement process of SAs pixels than No-SAs pixels. Meanwhile, Figure 5a also shows that the white area fraction (WA) of SAs pixels (0.64 ± 0.04) at full open state was much larger than No-SAs pixels (0.50 ± 0.01). The possible reason was that the thin oil film around the spacers after filling absorbs oil at the opening process further, leading to less oil left in the SAs pixels than No-SAs pixels.

The switching off of EWD devices was essentially the spontaneous spreading process of oil. Part of the oil climbs down the spacer first before its spreading and covering the open area for SAs pixels, while the oil directly spreads to the open area for No-SAs pixels (see inserted graphs showing in Figure 5b). The oil climbing down process induced by SAs brought extra time cost. On the other hand, according to the measured receding o/w contact angle difference for spacer (40°) and pixel wall (80°), we could carefully infer oil pinning stronger on SAs, which also hinders the spreading of oil. Therefore, introducing SAs did not show advantages in response time and optical uniformity of EWD switching off process.

## 4. Conclusions

Spacer arrays (SAs) on the junction of pixel walls were fabricated by a second photolithography process in this experiment. The introduction of SAs gave stronger mechanical strength of the electrowetting display (EWD) device. A relatively high density of the spacer array (1:4) was found the best density for mechanical strength, oil motion controllability, and optical performance of white area fraction considerations, because each spacer could only attract oil in the four pixels around this spacer. Enough high SA heights (60 μm and 40 μm) was necessary for oil moving to be a large droplet around spacer with high uniformity and also the high white area fraction, because of the increased oil aggregation area provided by long way in spacer. In this paper, spacer arrays were introduced to EWDs, leading to improved oil motion uniformity and white area fraction, and enhanced mechanical strength. Meanwhile, the switching with spacer array was reversible. Hence, this design of spacer array reasonably solved the contradiction between mechanical strength enhancement and optoelectronic performance in EWDs, providing potential applications in oil–water two-phase microfluidic devices.

## Supplementary Materials

The following are available online at https://www.mdpi.com/1424-8220/20/2/494/s1, Figure S1: The graphs showing the oil states of pixels with different SAs parameters with time at switching-on process.
