# Supplementary material for: Photolithography Fabricated Spacer Arrays Offering Mechanical Strengthening and Oil Motion Control in Electrowetting Displays
Source: Sensors (Basel). 2020 Jan 15;20(2):494. doi: 10.3390/s20020494 (PMC7014117; doi:10.3390/s20020494)
Supplement: Supplementary file 1 [file sensors-20-00494-s001.pdf]

Supplementary File

Photolithography fabricated spacer arrays offering mechanical strengthening and oil motion control in electrowetting displays

Yingying Dou [1,2], Lin Chen [1,2], Hui Li [1,2,3], Biao Tang [1,2,*], Alex Henzen [1,2] and Guofu Zhou [1,2,4,5,*]

1 Guangdong Provincial Key Laboratory of Optical Information Materials and Technology & Institute of Electronic Paper Displays, South China Academy of Advanced Optoelectronics, South China Normal University, Guangzhou 510006, P. R. China.

2 National Center for International Research on Green Optoelectronics, South China Normal University, Guangzhou 510006, P. R. China.

3 College of Mechatronics and Control Engineering, Shenzhen University, Nanhai Ave 3688, Shenzhen 518060, P. R. China.

4 Shenzhen Guohua Optoelectronics Tech. Co. Ltd. Shenzhen 518110, China.

5 Academy of Shenzhen Guohua Optoelectronics, Shenzhen 518110, China.

*Correspondence: tangbiao@scnu.edu.cn (B.T.); guofu.zhou@m.scnu.edu.cn (G.Z.)

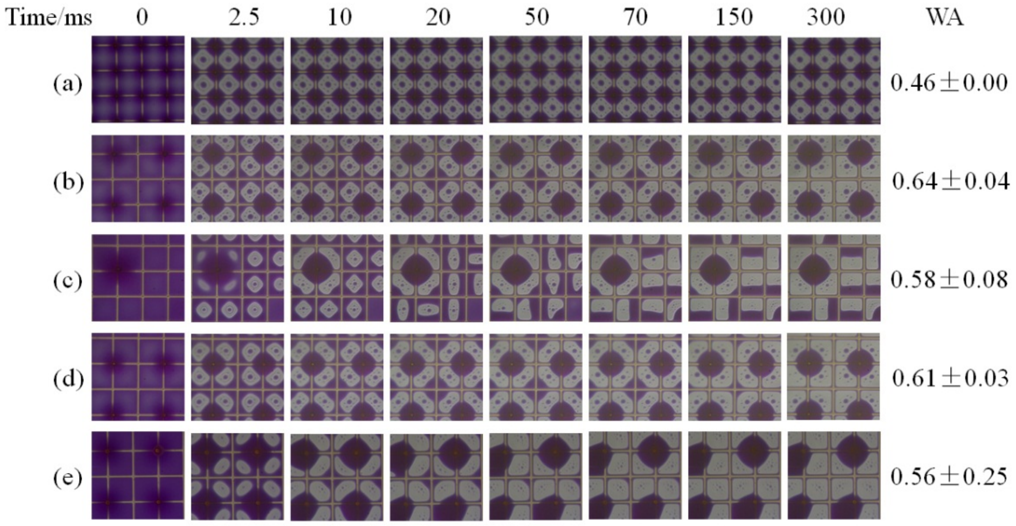

Figure S1. The graphs showing the oil states of pixels with different SAs parameters with time at switching-on process. 1) Effect of spacer densities (number of spacers: number of pixels, (**a**) 1:1, (**b**) 1:4 and (**c**) 1:16) on oil motion and gathering behavior with the spacer height of 60 μm. 2) Effect of spacer heights ((**b**) 60, (**d**) 40 and (**e**) 20 μm) on oil motion and gathering behavior with the spacer density of 1:4. WA is the White area fraction (WA) at 300 ms, while error bar is from ~8 data.
